# The Prevalence and Progression of Microvascular Complications and the Interaction With Ethnicity and Socioeconomic Status in People With Type 2 Diabetes: A Systematic Review and Meta-Analysis

**DOI:** 10.1155/jdr/3307594

**Published:** 2025-01-11

**Authors:** Thamer Alobaid, Janaka Karalliedde, Matthew DL O'Connell, Luigi Gnudi, Katie Sheehan, Ka Keat Lim, Salma Ayis

**Affiliations:** ^1^Department of Population Health Sciences, King's College London, London, UK; ^2^Cardiovascular Division, Faculty of Life Science and Medicine, King's College London, London, UK; ^3^Department of Health Services Research and Population Health Sciences, King's College London, London, UK; ^4^Department of Diabetes and Metabolic Medicine, King's College London, London, UK; ^5^Department of Health Services Research/Rehabilitation, King's College London, London, UK; ^6^Department of Health Economics, King's College London, London, UK; ^7^Department of Medical Statistics, King's College London, London, UK

## Abstract

**Introduction:** Diabetic nephropathy (DN) and diabetic retinopathy (DR) are serious complications of type 2 diabetes mellitus (T2DM). The reported estimates of prevalence and progression of DN and DR vary widely across studies. We undertook a systematic review and meta-analysis to determine the extent to which these variations in prevalence and progression of DN and DR may relate to different ethnic groups and socioeconomic status (SES).

**Methods:** We searched the databases Ovid MEDLINE, Global Health, APA Psych Info, Embase, and PubMed for publications from 2005 to September 2023, based on T2DM and DN or DR, which included patient's ethnicities and SES. Prevalence estimates were summarized by meta-analysis using random effects models for each microvascular complication, stratified by ethnicity and SES. Data on progression was summarized narratively.

**Results:** Twenty-seven studies were included. The overall prevalence of DN was 18% (95% CI: 14%, 22%) with no differences noted by ethnic group. Low economic status and low education levels were associated with a 4% increased risk of DN compared to higher levels. Higher prevalence of DR was noted among the Afro-Caribbeans, 28% (95% CI: 11%, 46%), compared to the White/Caucasian 19% (95% CI: 11%, 27%), and Asian/Indo Asians 25% (95% CI: 9%, 41%). Low-SES populations have a higher prevalence of DR than high-SES populations. The average prevalence was 16% (95% CI: 11%, 22%) among the high economic status group, compared to 25% (95% CI:20%, 30%) for the low economic status. Our study showed that Black ethnicity was associated with a higher risk of progression to end-stage renal disease (ESRD) and diabetic maculopathy compared to other ethnicities. People with high SES had a lower rate of DR progression than those with low SES, odds ratio (OR) (0.63, 95% CI: 53%, 74%).

**Conclusion:** Ethnicity and SES may be associated with differential risk of development and progression of DN and DR. The available evidence was limited by the number of studies and small samples for certain ethnic/socioeconomic groups.

## 1. Introduction

Diabetes is a global public health problem. People with diabetes are at substantial risk of developing microvascular complications including nephropathy and retinopathy [1]. The prevalence of diabetes has been rising rapidly in the last few years globally [1]. Type 2 diabetes is commonly associated with adults but is increasing in the younger age group which accounts for more than 90% of all diabetes globally [2]. Complications of diabetes are divided into microvascular and macrovascular. The commonest types of microvascular complications are diabetic kidney disease (diabetes nephropathy (DN)) and diabetes eye disease (diabetes retinopathy(DR)) [3].

Several environmental and biological factors contribute to these complications including age, uncontrolled blood pressure, and high HbA1C [4]. Ethnicity and socioeconomic status (SES) are two additional factors that could have a role in developing microvascular complications [5]. Many studies have shown associations between ethnicity and microvascular complications [6]. The findings of these are, however, inconsistent and estimates vary widely. Some studies showed that Black ethnicity participants have a higher tendency to develop microvascular complications [7]; others showed no differences between ethnicities [4, 7, 8]. Moreover, few studies have suggested that low SES is associated with an increased risk of microvascular complications [9]. In this study, we use the term “socioeconomic status” to refer to a combination of education, income, and occupation levels. “Economic status” specifically refers to the financial aspect of SES. While both are related, they capture different dimensions of a person's social and economic standing.

In this systematic review and meta-analysis, we synthesized the available evidence on the prevalence of microvascular complications among populations of different SES and ethnicity. We also narratively presented studies that have looked at the progression of these complications, focusing on the two outcomes: DN and DR.

This study is aimed at, first, reviewing and summarizing the estimates of the prevalence of DN and DR among different ethnicities and SES as reported in the current literature in studies that provide estimates by ethnicity; second, examining and synthesizing evidence on predictors of progression of these two microvascular complications, similarly by ethnic groups and for various SES in people with type 2 diabetes.

## 2. Methods

### 2.1. Protocol and Registration

The protocol for this systematic review and the meta-analysis was registered on the International Prospective Register of Systematic Reviews (PROSPERO: CRD42022306899).

The guidelines for Preferred Reporting Items for Systematic Reviews and Meta-Analyses (PRISMA) were followed. Covidence software was used for deduplication, title and abstract screening, full-text review, data extraction, and the development of PRISMA charts.

The Population, Index Prognostic factors, Comparator/Prognostic factors, Outcomes, Timing and Study type (PICOTS) framework was used for reviewing the papers about prevalence and predictors of progression [10]. No geographical limits were used for our search. Studies published in English between January 2005 and September 2023 were considered by the review.

### 2.2. Search Methods and Data Sources

We searched the databases: Ovid MEDLINE, Global Health, APA Psych Info, Embase, and PubMed. The forward citation tool in the Web of Science was used, and all papers that cited those included in the review were also considered.

We employed search terms for the population (type 2 diabetes that have addressed ethnicity, SES, or both) and outcomes (prevalence and progression for microvascular complications, nephropathy, and retinopathy).

The inclusion criteria were English language papers, full peer-reviewed articles since the abstract alone may not be useful and may not have enough data, and studies about people with type 2 diabetes and microvascular complications (nephropathy and retinopathy) that have addressed either ethnicity, SES, or both. The exclusion criteria are papers that do not fit the inclusion criteria.

We included published randomized controlled trials (RCTs) and observational studies that estimated the prevalence and/or progression of DN and/or DR which also specified the estimates by ethnicity and/or SES. Among the papers selected for this review, SES was defined by educational level in some studies, while in others it was defined by the economic status. Due to the limited number of studies available on DN and SES (*n* = 2), a formal meta-analysis was not conducted for this subgroup. Instead, the results are presented narratively. DN was defined as persistent proteinuria with a low estimated glomerular filtration rate (eGFR < 60 mL/min/1.73 m^2^) in people with diabetes [11].

DR definition was based on the classification of findings on a dilated funduscopic examination [3]. Abnormalities can be broadly divided into proliferative diabetes retinopathy (PDR), nonproliferative diabetes retinopathy (NPDR), maculopathy (M), and photocoagulation (P) [3, 12], each of which has further refined stages [3, 13].

Progression for DN was defined as the worsening of kidney function till it reached ESRD. Retinopathy progression is defined as the worsening of disease from one stage to another till it reaches the final stages [5]. For DN, all studies that examined the progression/remission of albuminuria eGFR decline or the progress to ESRD were included. For DR, all studies that included worsening of the retinopathy to reach a further stage of the disease, including the final stages of M or P, were included [12]. M is the stage where the damage occurs at the macula whereas, the P stage is where laser therapy is performed on the retina and leads to shrinkage and scarring of the abnormal blood vessels [3]. Studies suggested that 7.2% of people with DR progress to M [3].

### 2.3. Data Collection Process

Two reviewers (T.A. and S.A.) independently screened titles and abstracts using the eligibility criteria and identified papers for inclusion. From the selected papers, the full text was screened against the eligibility criteria, and the final papers to be included in the review were identified. Conflicts were resolved by consensus.

The first author summarized the included studies using the template built into the Covidence software and included the following: the study ID, title, lead author's contact details, journal name, abstract, the country in which the study was conducted, methods, the aim of the study, study design, participants descriptions, total number of participants, the tables that describe the basic demographic characteristics, and the main results.

### 2.4. Statistical Analysis

Estimates of prevalence and progression of microvascular complications by ethnicity and SES for each of the outcomes, nephropathy and retinopathy, were summarized in the tables. Prevalence data were meta-analyzed using random effects models [14]. Both observational and RCTs were presented using forest plots stratified by study design, ethnicity, or SES. We measured the heterogeneity of the studies using *I*^2^ statistics which describes the percentage of variation throughout studies that is due to heterogeneity rather than chance [15]. Where prevalence estimates were reported for different and independent samples, these were analyzed as such and were treated as separate groups. We used Stata software (v.17) to perform our statistical analysis. Studies on progression were narratively presented [14].

### 2.5. Quality Assessment

The quality assessment for the observational studies was completed using the Meta-analysis of Observational Studies in Epidemiology (MOOSE) [16]. For RCTs, we used the Cochrane Risk of Bias Tool 1 [17].

## 3. Results

**Figure 1:**
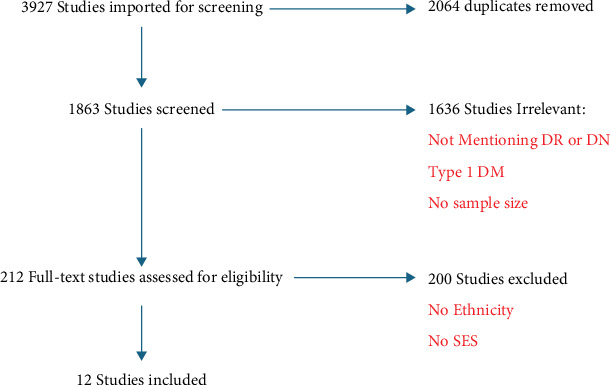
PRISMA chart of the studies that are selected for the systematic review.

According to our search strategy, 3927 studies were imported to Covidence software for screening. Two thousand and sixty-four duplicates were removed, and 1863 studies were screened by their abstract for suitability based on our eligibility criteria. Out of these, 1636 were excluded as irrelevant due to several reasons such as no mention of ethnicity or SES or no sample size was given. By full text, we assessed 227 studies, out of which 200 studies were excluded due to one or more of the exclusion criteria mentioned earlier. The final selection resulted in 27 studies and data extraction, the quality check was made on these, and meta-analyses were performed on 13 studies (Figure 1). Of the 13 studies included in the meta-analysis, six were from the United Kingdom, three from the United States, two from Japan, one from South Korea, and one from Australia. The prevalence estimates were obtained from highly heterogeneous settings, as the definition of SES varied across studies, and the age distributions differed significantly. This variability may affect the reliability of the pooled estimates.

### 3.1. DN and Ethnicity

**Figure 2:**
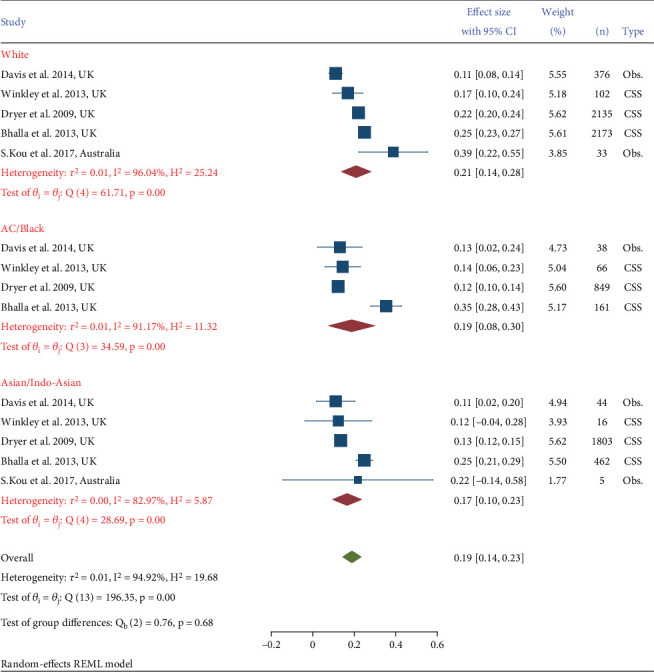
Prevalence of diabetic nephropathy in different ethnicities and study types.

Five studies provided data on nephropathy and ethnicity [8, 11, 18, 19]. Three of these were cross-sectional studies and one was a prospective observational cohort study. Ethnicities included were White/Caucasian, Black/Afro Caribbean (AC), and Asian/Indo-Asian (IA)/South Asian (SA). The pooled estimates by ethnicity (Figure 2) showed a similar prevalence of DN in each of the ethnic groups considered. Nephropathy was measured by microalbuminuria in studies by Winkley and Thomas [18] and Davis [8], whereas in Dreyer et al.'s [19] and Bhalla et al.'s [11] nephropathy was measured by eGFR. For Winkley and Thomas' study, the prevalence among White (prevalence 17%, 95% CI: 10%–24%) was higher than other groups; Black (prevalence 13%, 95% CI: 6%–23%), and Asians (prevalence 12%, 95% CI: 4%–28%). In Davis' study, the prevalence was higher among the Black group (prevalence 13%, 95% CI: 0.02–0.24) than the White (prevalence 11%, 95% CI: 8%–14%) and the Asian (prevalence 11%, 95% CI: 2%–20%) groups. Bhalla et al. [11] also showed that Black ethnicity has a higher prevalence compared to others (prevalence 35%, 95% CI: 28%–43%). In addition, Kou et al. [20] which compared the White and Asians showed that White ethnicity has a higher prevalence of developing DN (prevalence 39%, 95% CI: 22%–55%) than Asians (prevalence 22%, 95% CI: 14%–58%). Figure 2 shows the results from these studies classified by ethnicity and study type.

### 3.2. DN and SES

Only two studies were identified examining DN and SES, and these are presented narratively rather than as part of a meta-analysis due to the insufficient number of studies. Two studies classified people with type 2 diabetes into high and low economic levels and high and low educational levels [21, 22]. Kim et al. divided the population further by sex. A high educational level indicates a college degree or above, whereas a low education level indicates less than a high school education. The economic level was determined by annual income. Each study employed different cutoff points for income measured in US dollars.

**Figure 3:**
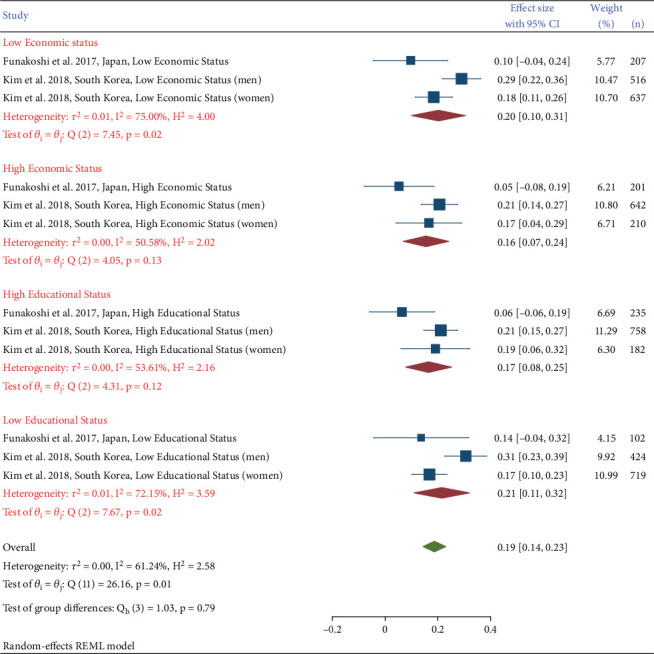
Prevalence of diabetic nephropathy in different socioeconomic statuses.

Groups with high economic and educational status had a lower prevalence of DN than groups with low economic and educational status. In Kim et al. [21], the prevalence of DN was higher in lower economic and educational level groups. The study also showed that prevalence was higher among males in low economic status (prevalence 29%, 95% CI: 22%–36%) than females whose prevalence was 18% (95% CI: 11%–26%), and similarly higher in male than female in low educational level, with a prevalence of 31% (95% CI: 23%–39%) and 17% (95% CI: 10%–23%), respectively (Figure 3). In Funakoshi's study [23], people with type 2 diabetes and low educational level and low economic status had a higher prevalence of developing DN (prevalence 14%, 95% CI: 4%–32%), compared to people with higher economic and educational levels (prevalence 10%, 95% CI: 4%–24%).

### 3.3. Retinopathy and Ethnicity

**Figure 4:**
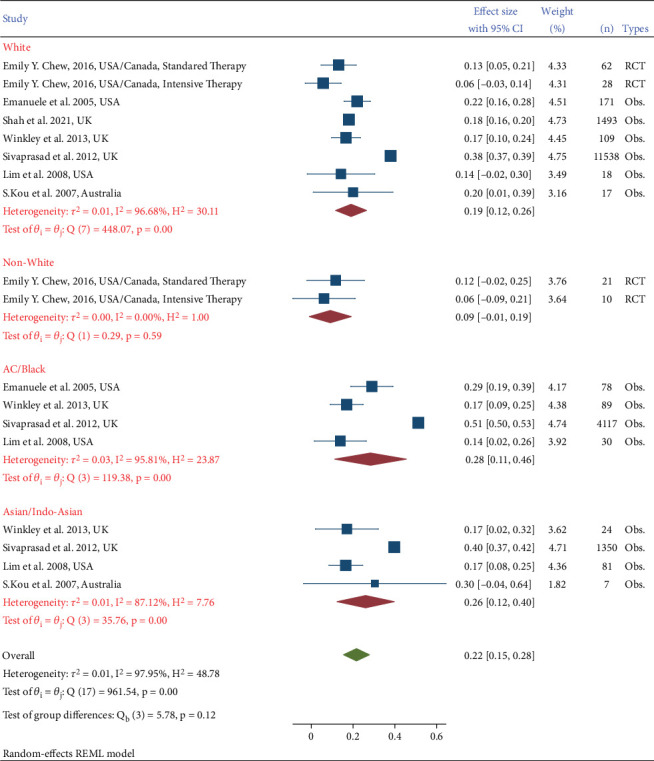
Prevalence of diabetic retinopathy in different ethnicities and study types.

Seven studies provided data on retinopathy and ethnicity. The prevalence among White people was 19% (95% CI: 12%, 26%) while among Black ethnicity, it was 28% (95% CI: 11%–46%). Moreover, for Asian ethnicity, the prevalence estimates were 26% (95% CI: 12%–40%). Chew [24] studied two groups, White and non-White, where the treatments were standard or intensive, whereas Shah et al.'s study [12] included only White ethnicity. Most studies showed a higher prevalence of DR in people with Black ethnicity compared to other ethnicities. While Winkley and Thomas' study showed no differences, Lim et al.'s [25] and Kou et al.'s study [20] showed a higher prevalence among Asians compared to other ethnicities. Higher prevalence was shown in observational studies compared to RCTs in all ethnicities, and the prevalence was higher among the Black and the Asian compared to the White group. Overall, there are differences across subgroups and heterogeneity was high within most of this, while the overall heterogeneity was modest (Figure 4).

### 3.4. Retinopathy and SES

**Figure 5:**
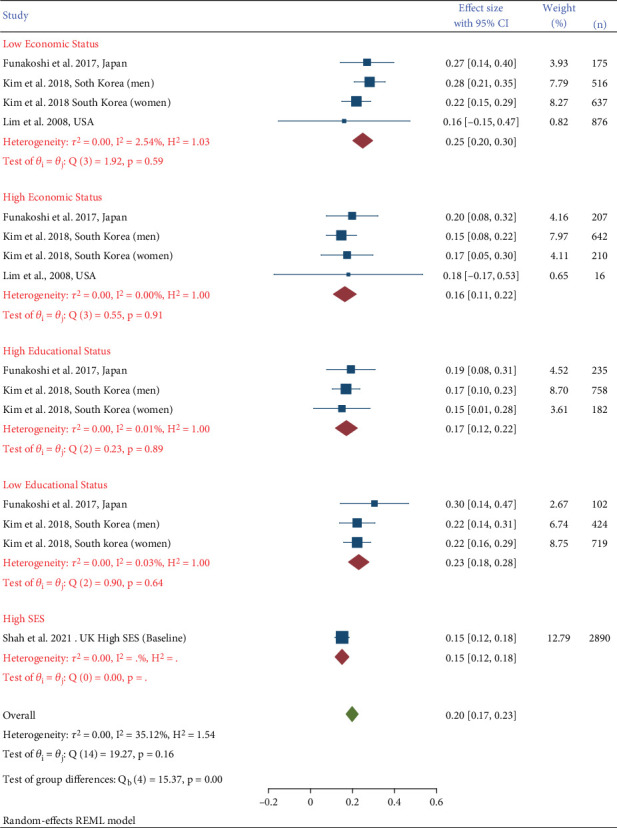
Prevalence of diabetic retinopathy in different socioeconomic statuses.

There were four studies that were stratified according to the economic and/or educational level [12, 21, 23, 25].  Figure 5 shows that lower economic status populations have a higher prevalence of DR than high economic status populations. The average prevalence was 16% among the high economic status group, compared to 25% for the low economic status group. Similarly, high educational level studies showed a prevalence of 17% compared to 23% for low educational level studies.

### 3.5. The Progression of Nephropathy and Retinopathy

#### 3.5.1. DN

Studies vary in how progression was defined, but mostly it was defined as a decline in eGFR level. Dreyer et al.'s study [19] showed that the risk of DN progression among AC and SA ethnicities is lower than that in the White; odds ratios (ORs) after adjusting for other risk factors were OR 0.49 (95% CI: 0.43–0.57) and OR 0.79 (95% CI: 0.71–0.78) [19], respectively. The same study showed that AC and SA ethnicities have a higher risk for progressing to ESRD compared to White ethnicity (OR 1.39 (95% CI: 1.06–1.81) and OR 1.54 (95% CI: 1.26–1.88) for the two groups, respectively).

#### 3.5.2. DR

Chew followed people with DR for 8 years and reported a reduced risk of progression in those who were on intensive glycemic control compared to those on standard glycemic control (adjusted hazard ratio (HR) 0.45 (95% CI: 0.32–0.64)) [6]. Furthermore, White participants were at a lower risk compared to non-White (OR 0.48 (95% CI: 0.22–1.07)).

In Shah et al.'s study, the 7-year follow-up for DR noted people with type 2 diabetes and White ethnicity had a nonsignificant lower rate for DR progression with OR 0.96 (95% CI: 0.80–1.14). Moreover, people with high SES had lower ORs of progression than those with low SES (OR 0.63, 95% CI: 0.53–0.74). The OR for sight-threatening retinopathy was also lower in the White than in other ethnicities (OR 0.71, 95% CI: 0.50–1.03), yet insignificant. Similarly, high SES was associated with a lower but insignificant risk of DR progression (OR 0.84, 95% CI: 0.62–1.13).

## 4. Discussion

Our results have shown wide variability in the prevalence of diabetic complications (DR and DN). From our synthesis, no clear ethnic differences were found for DN, but substantial heterogeneity was noted between studies. For example, the range of DR was between 14% and 35% in two UK studies (Winkley and Thomas and Davis) [8, 18]. Similar discrepancies were noted among the White and the Asian groups.

In agreement with the traditional established risk factors, older age and diabetes duration were associated with increased risk of microvascular complications. A study by Baskar et al. reported that the duration of diabetes is related to the development of microvascular complications in general, especially in the Black population with OR 1.09 (95% CI: 1.08–1.11) [4] comparing to White ethnicity group.

While for DN no ethnic differences in the overall prevalence were found, for DR, higher prevalence was seen among the AC (28%) and the Asian (25%) compared to the White/Caucasian (19%). For both complications, higher prevalence was noted among low economic levels and low SES. For DN, for example, the prevalence was 20% and 16% among the high and low economic status, respectively, and for DR the corresponding figures were 25% and 16%.

While our summary suggests no differences across ethnicities in the prevalence of DN, however, the literature has many controversial findings. For example, a review by Davis, 2008, explaining the UK Prospective Diabetes Study (UKPDS), showed that IA and White have a higher risk of developing nephropathy than AC [8]. Adjusted HR for microalbuminuria, macroalbuminuria, and a creatinine clearance (CrCl) ≤ 60 mL/min/1.73 m^2^, was used to assess DN, whereas IA ethnicity was associated with twice the risk of the White ethnicity.

The heterogeneity across the included studies, particularly in the definitions of SES and variations in age distribution, may limit the comparability of the results. This should be taken into account when interpreting the findings. Within the included literature, SES was defined as economic and educational level. In some studies, the economic level was measured by the income rate, whereas the high educational level indicates college level and above [26]. Some studies suggest that both lower educational and economic levels result in a higher prevalence of DR and DN [27]. Few studies have shown associations between low SES and microvascular complications using different measures for SES, including income and education [28, 29]. Sharma et al. estimated the prevalence of microvascular complications among low educational level at 23.6% compared to 14.7% among those with higher levels [30].

While several studies have shown a higher prevalence of microvascular complications among more deprived people compared to less deprived, there are nonetheless, few studies that showed minimal difference among those with various levels of education.

Regarding a progression of DN in different ethnicities, Ali et al. [7] showed that AC declined faster in their eGFR compared to White and SA, with an annual decline rate of −2.12 mL/min/1.73 m^2^ [5]. Mathur et al. showed also that AC has a slower rate of progression of eGFR, −0.55 (95% CI: −0.61 to −0.48) mL/min/1.73 m^2^ compared to the White −0.64 (95% CI: −0.68 to −0.60), while faster rate of decline was shown for the SA −0.77 (95% CI: −0.81 to −0.74) mL/min/1.73 m^2^; all of which are lower than rapid decline (defined as eGFR loss of > 3.0 mL/min/1.73 m^2^ per year) [31–33]. Moreover, another study by Mathur et al. in the East London database showed that White ethnicity has a higher rate (prevalence 7.4%) of developing any microvascular complications compared to Black and SA ethnicities (prevalence 5.8%, and 3.4%, respectively) [34].

On the other hand, for DR progression, a study by Drew showed that Black ethnicity has an increased risk, OR 1.14 (95% CI: 0.67, 1.94), for progression of retinopathy compared to the Hispanic population while White ethnicity has a lower risk of progression (OR 0.50, 95% CI: 0.24–1.05) [35] compared to Hispanic population, too.

Our findings have shown that overall, ethnicity and SES have a role in the prevalence and progression of microvascular complications. The interaction of these factors, and the association with other confounders such as age, and diabetes duration remains complex and not clear how to disentangle an independent contribution for each. To better understand the underlying mechanisms and patterns of such associations and their role in the development of microvascular complications, further investigations, with larger samples, and long-term follow-up might help.

This evidence synthesis provides a summary of the prevalence of two major microvascular complications, nephropathy and retinopathy, that are common among people with type 2 diabetes. The study supplements available evidence on the burden of diabetes and the increased risk among racial and ethnic minority populations and disadvantaged SES in various parts of the world. An elevated level of heterogeneity across studies was seen. RCTs tend to provide lower prevalence, compared to observational studies; this might be interpreted as due to the selective nature of RCTs that is often reported.

The study findings emphasized the vital need to focus on primary prevention to address the current and expected increase in the prevalence of diabetes, and the consequent increase in cardiovascular complications among vulnerable populations. Around 40% of the participants studied have either DR or DN, and the burden among the minority and socially disadvantaged populations was higher. Thus, the study may be of help to clinical and population-based strategies, aiming for prevention, timely screening, early identification, and treatment, for more efficient outcomes.

### 4.1. Strengths and Limitations

To our knowledge, this is the first systematic review that compiled the evidence on the prevalence and progression of two important microvascular complications and their interaction with ethnicity and SES in people with type 2 diabetes. The study supplements available evidence on the increased risk of nephropathy and retinopathy among certain ethnic minority and socially disadvantaged groups.

On the limitation side, a major limitation of this study is the heterogeneity of the included studies, particularly in terms of SES definitions and age groupings, which complicates the synthesis of findings. The elevated level of heterogeneity between studies suggests that different methods, study designs, sample sizes, and different adjustments for various confounders may have contributed to these differences, while some remain unexplained. Lack of standardization, age, diabetes duration, and the level of the disease in terms of severity and eGFR at baseline indicate that the summary estimates need to be cautiously interpreted. For the DN studies, we have a limitation in the CKD progression in SES. Here, we excluded CSS and that reduced the heterogeneity/or did not affect the heterogeneity or the summary estimate. The study also draws attention to the limited number of studies that provide estimates by ethnicity. While most samples represent a composite of different ethnic groups, estimates of prevalence and progression by ethnicity were only provided by a small number of studies. The definition of SES varies across studies, as some studies used economic status and others used educational level as indicators of SES, but these were treated separately rather than combined which meets the definition of SES, which would impact the resulting classification [36]. A formal test for publication bias was not conducted in this study, and the potential for such bias remains a limitation.

## 5. Conclusion

In general, there are many microvascular complications in type 2 diabetes mellitus, but our study focused on DN and DR. Many factors are involved somehow and have shown an association with these complications including ethnicity and SES. From the evidence compiled in this study, it was clear that the interactions between ethnicity, SES, and other confounders, such as age, gender, disease duration, and severity, are complex and not well investigated in the literature to date. Evidence from this systematic review and meta-analysis, and lessons learnt from the quality of studies, will be taken forward to improve estimates in future investigations that will be part of a comprehensive thesis. The plan is to find out more about the interactions between ethnicity and SES alongside other confounders and their role in the prevalence and progression of microvascular complications. Large cohorts of electronic health records (EHRs) of multiethnic groups will be employed in these planned investigations.

## Data Availability

The data that support the findings of this study are available from the corresponding author upon reasonable request.
